# Direct to psychology for sleep disorders: Innovating models of care in the hospital and health service

**DOI:** 10.1177/13591053241267272

**Published:** 2024-08-05

**Authors:** Sara Winter, Sara Crocker, Tricia Rolls, Deanne Curtin, Jessica Haratsis, Irene Szollosi

**Affiliations:** 1 The Prince Charles Hospital, Australia; 2 University of Queensland, Australia; 3 Metro South Addiction and Mental Health Services, Australia

**Keywords:** sleep, sleep disorders, insomnia, OSA, obstructive sleep apnoea, allied health led, direct to psychology, sleep psychology, health psychology, behavioural sleep medicine, model of care re-design

## Abstract

A ‘Direct to Psychology Insomnia’ pathway was developed for implementation within a multidisciplinary sleep disorders service in a tertiary hospital in Brisbane, Australia. The project was informed by implementation science principles and methodology to re-design the model of care (MoC). A consensus group workshop using the Nominal Group Technique (NGT) with 12 multidisciplinary staff was undertaken to develop the new MoC. The workshop explored inclusion and exclusion criteria for a Direct to Psychology pathway including patient flow and enablers. The team endorsed a MoC that was acceptable to stakeholders and addressed service-level imperatives. The findings highlighted that patient inclusion or exclusion should be overseen by the Sleep Physician team and an Advanced Psychologist with behavioural sleep medicine expertise. Continuum of care for patients referred via primary care providers was considered. Barriers and risks to the MoC changes were identified which informed the refinement of the MoC.

## Introduction

Insomnia is a common health complaint, resulting in adverse health consequences and significant societal and economic impacts due to lost productivity, vehicle and equipment accidents, and work absenteeism (Deloitte Access Economics, 2021; Natsky et al., 2020). Currently, most individuals who are diagnosed with insomnia are prescribed pharmacological treatments as a first-line intervention via their primary healthcare professionals, which have suboptimal long-term efficacy and implications for health and dependency (Miller et al., 2017). Cognitive behaviour therapy for insomnia (CBT-I) is the gold-standard treatment for insomnia (Qaseem et al., 2016; Ree et al., 2017) though access to evidence-based programs and practitioners with sufficient behavioural sleep medicine skill is a significant challenge (Meaklim et al., 2021). Sweetman and colleagues’ research investigated the implications of the adoption of psychological interventions as an appropriate treatment for patients with Co-Morbid Insomnia and Obstructive Sleep Apnoea (COMISA; Sweetman et al., 2017) to improve obstructive sleep apnoea (OSA) therapy outcomes. Their 2019 RCT investigated the effect of cognitive behavioural therapy for insomnia (CBTi) on OSA severity and found that a four-session CBTi intervention improved sleep consolidation and promoted a 15% decrease in OSA severity in COMISA patients (Sweetman et al., 2019, 2023).

In a major public multidisciplinary sleep centre in Brisbane, Australia, patients are commonly referred for excessive daytime sleepiness or suspected Obstructive Sleep Apnoea (OSA). The referral may indicate a primary or comorbid insomnia that would benefit from psychological intervention. The existing pathway to access psychology intervention for insomnia is via the Sleep Physician, yet the under-pressure nature of tertiary healthcare waitlists often means patients have a significant wait time between initial referral, Sleep Physician consultation, and subsequent access to psychology treatment, further compounding their insomnia and contributing to health and societal consequences. In most straightforward cases, insomnia symptomology often resolves after specialised psychology intervention (excluding confounding medical factors), meaning that patients are often waiting longer than necessary for insomnia management. Conversely, not all insomnia presentations are straightforward, and along with limited referral information, this may mean that medical review either pre- or post-psychology intervention may be warranted in some circumstances. Within the public health setting, challenges in servicing ever-growing and ageing populations, particularly in specialist outpatient settings, have been a long-term issue (Stute et al., 2018). This is compounded by the ongoing impacts of the COVID-19 pandemic, with demands for specialist outpatient services continuing to increase relative to the resources available to meet these needs. These demands within our health services require innovations in healthcare delivery to maintain service sustainability and meet patient health outcomes (Mutsekwa et al., 2022; Stute et al., 2018).

### Direct to allied models of care

There is precedent across healthcare settings for variations of ‘Direct to’, ‘Allied Health-first contact’ and ‘Allied Health-led’ Models of Care (MoC) (Goodman et al., 2018; Stute et al., 2018). Stute and colleagues (2018) found that allied health first clinics result in high patient, referrer, and consultant satisfaction, and are a cost-effective management strategy for waitlist demand. Goodman and colleagues (2018) identified several determinants of success for allied health-led models of care including treatment adherence, treatment effectiveness, safety, and appropriateness of care. These determinants are translatable factors across all allied health disciplines, but particularly within insomnia management via psychology intervention as it relies heavily on patient engagement with treatment to optimise patient outcomes.

Our study aimed to build on existing literature and developed a ‘Direct to Psychology’ MoC with reference to the local context in a major public hospital multidisciplinary sleep service using implementation science methodology (Glasgow et al., 2019). Subsequent aims are to explore the feasibility and viability of implementing the new MoC within existing resources. This MoC aims to improve service efficiency by implementing a direct Psychology pathway for appropriate insomnia presentations which bypasses long waits for specialist medical review. The MoC targets consumers with highly prevalent, non-complex insomnia to directly access the appropriate clinicians with the behavioural sleep medicine expertise to optimize their treatment. We expected that the MoC would be acceptable to multidisciplinary stakeholders and that any identified barriers and risks of a ‘direct to’ MoC would be addressed within the model. We expect that the model will ultimately improve patient experience and outcomes as well as service level outcomes of timeliness of access to evidence-based care (to be undertaken in future work). We also expect that upon demonstration of the safety and efficacy of the model, the MoC could form the basis of future psychology-led service provision work across other services through translational research.

## Method

This project has received ethics approval via the LNR pathway with a waiver of informed consent with the Darling Downs Health Human Research Ethics Committee (HREC 2022 QTDD 88350). The Protocol is listed with the Australian and New Zealand Clinical Trials Registry (ACTRN12622001086752).

### Participants

Participants were multidisciplinary clinical team members of The Prince Charles Hospital Sleep Disorders Centre. All multidisciplinary staff working within the service at the time of undertaking the consensus group workshop were invited to participate. Participant group demographics included sleep physicians, sleep scientists, and psychologists (full demographic breakdown of participants is presented in Table 1 in Supplemental Item).

### Procedure

The project proceeded in two phases; Phase 1 involved a Consensus workshop, and Phase 2 consisted of a collaborative in-service to present the final model and plan for implementation.

#### Phase 1: Consensus workshop

A consensus group workshop using the Nominal Group Technique (NGT; Delbecq et al., 1975; McMillan et al., 2016) was undertaken in November 2022, with all multidisciplinary clinical team members invited to participate. The workshop was of 1 hour duration and was proactively facilitated by an experienced clinician (SW) to ensure participants adhered to the NGT process. The purpose of the workshop was to elicit opinions on the proposed Direct to Psychology model based on preliminary work by Krebs and Ellender (2021), including initial inclusion and exclusion criteria, and to gather expert advice on how this model should be modified for the local context including potential risks and benefits. The overarching aim was then to reach a consensus on the Direct to Psychology model for implementation.

The workshop was completed as a hybrid meeting with participants present online (Microsoft Teams) as well as in person. Five questions were sent to participants in advance regarding inclusion and exclusion criteria, patient flow, benefits, and risks. Participants were advised that a Direct to Psychology Service Model was being considered, utilising inclusion and exclusion criteria proposed by Krebs and Ellender (2021). Participants in the workshop were asked several questions to elicit perspectives (see Supplemental Item 1.0).

The five questions were answered by participants in four stages (Delbecq et al., 1975; McMillan et al., 2016): Stage 1: Silent Generation; Stage 2: Round Robin; Stage 3: Clarification; Stage 4: Summarising and Voting. These stages are described in detail in Supplemental Item 2.0.

#### Phase 2: Presentation of final models

After analysis of the workshop outcomes on themes that emerged from participant responses as well as voting on preferred service models, feedback on workshop outcomes was presented via a collaborative in-service presentation in February 2023 to the clinical team before implementation of the service change. The final model was presented to the multidisciplinary team, and any final questions or clarifications were voiced, and planning for implementation was discussed.

### Data analysis

Participant demographic details, as well as voting outcomes, are described using frequencies, percentages and range as applicable. Quantitative data collected via the Consensus group workshop for confidential responses produced during the silent generation phase were evaluated utilising inductive Thematic Analysis (TA) (Braun and Clarke, 2006; Xu and Zammit, 2020). TA is a qualitative analytic method for identifying, analysing, and reporting patterns (themes) within data, and is complementary to consensus group methods such as the NGT in the analysis of the individual and grouped themes generated during the group interactions.

Data was coded using iterative steps to ensure a robust approach, including (1) line-by-line verbatim transcription of the confidential responses from the Silent Generation phase across all five questions by SW) responses were separated into succinct/separate ideas of a few words or a sentence depending on the nature of the response by SW. Where a response had a few embedded ideas in the sentence, these ideas were separated for individual analysis; (3) independent data immersion by two independent coders (SC, TR) into data and line-by-line coding; (4) identification of preliminary themes and identification of supporting quotes; (5) review of generated themes and quotes by SW to refine and decide on final themes and quotes.

Two psychologists (SC, TR) independently reviewed the transcribed data using an inductive approach to uncover themes. Instructions to the Coders and approach to the analysis are described in Supplemental Item 3.0. Coding was performed manually. The decision was taken not to use software to analyse the data (such as NVivo) due to the relatively small data set.

Ideas generated and grouped through team agreement during the Round Robin and Clarification stages of the workshop in reference to questions 1–3 were transcribed during the workshop. These were not subjected to TA as these ideas were already synthesised by the group during the NGT process. The synthesised themes generated during the *Round Robin* and *Clarification* phases are summarised in the Results section.

## Results

### Phase 1: Consensus groups

#### Thematic analysis of silent generation responses

The key themes and sub-themes that emerged from the silent generation stage of the consensus group workshop are presented in Table 1. Each theme and subtheme was presented in order of preponderance of responses to indicate the strength of each theme. However, it should be noted that the importance placed on each theme was also considered in light of the consensus group voting.

**Table 1.** Themes and sub-themes from the Consensus Group Workshop.

Workshop grouping question	Themes and subthemes
Inclusion criteria	Insomnia disorder present/Suspected
	High Insomnia Severity Index
	Distress/Impairment
	Other relevant comorbidities
	‘Other’ Considerations
Exclusion criteria	Significant comorbidities
	Sleep disordered breathing Complex health/neuromuscular conditions
	Other sleep disorders
	Complex mental health
	‘Other’ Presenting Issues/Impressions
Patient flow through the service	Psychologist first pathway
	Flexible pathways
	Concurrent pathway Medical Review First Pathway
	‘Other’ Considerations
Benefits	Service efficiencies
	Streamlined processes
	Reduced physician demands
	Improved patient outcomes
	Increased access/timely care Tailored/patient centred care
Risks	Gaps in assessment/treatment
	Missed medical diagnoses
	Not enough information for appropriate triage
	Reduced access/efficiencies
	Slower access to medical treatment
	Increased psychology demand
	‘Other’ Potential Risks
	No risks

The themes that emerged are presented in Table 1 relative to the five grouping questions. Representative de-identified supporting quotes are provided for each theme and subtheme for illustrative purposes. Each quote is identified by a response number representing the order that the response was transcribed into the larger dataset.

### Inclusion criteria

When considering appropriate *Inclusion Criteria*, three themes emerged: Insomnia Disorder Present/Suspected, Other Relevant Comorbidities, and ‘Other’ Considerations.

#### Insomnia disorder present/Suspected

A key theme around inclusion criteria for referral to a Direct to Psychology pathway from triage was the presence of insomnia disorder or suspected in the written referral.

Clinical criteria for duration and frequency of disordered sleep experience/insomnia (4)

Referral indicates main referral reason is primary insomnia (11)

This theme fell into two main subthemes: high insomnia severity index and distress/impairment.

#### High insomnia severity index

Participant responses indicated a preference for quantifying insomnia disorder as elevated scores (i.e. in the clinical range) on the Insomnia Severity Index (ISI), a common measure of insomnia symptoms used clinically and in the empirical literature.

ISI> given threshold (9)

ISI> 15 if available (21)

#### Distress/impairment

Participants also indicated that the insomnia experience should be causing distress and/or impairment, warranting Psychology intervention.

Associated impairment (function) and distress at clinical levels (5)

#### Other relevant comorbidities

Participants identified other psychological and behavioural presentations relevant for Direct to Psychology referral, including the presence of anxiety, Continuous Positive Airway Pressure (CPAP) treatment/behavioural treatment adherence concerns, and the presence of sleep-disordered breathing where there is also a high likelihood of insomnia (high ISI).

(a query of) anxiety (10)

Treatment adherence to CPAP/suboptimal adherence (6)

Keep Category 2 and 3 even if AHI > 30^1^ *if* ISI is high (43)^2^

#### ‘Other’ considerations

In response to the question of inclusion criteria for the Direct to Psychology Pathway, the final theme to emerge was ‘Other’ Considerations for entry to this pathway, which included patient consent to the triage decision to direct the patient to the Psychology pathway, and relapse prevention.

Pt [patient]consents to psychology assessment and investigation (16)

(a query of) prior sleep psychology input (i.e. for relapse management) (17)

### Exclusion criteria

When considering appropriate *Exclusion Criteria*, two themes emerged: Significant Comorbidities, and ‘Other’ Presenting Issues/Impressions.

#### No significant comorbidities

A key theme around exclusion criteria, noted by a majority of respondents, was that the presence of significant comorbidities should be a key consideration for exclusion from the Direct to Psychology Pathway from triage (i.e. before seeing the Sleep Physician).

Medically complex co-morbidity, including neurodivergent diagnosis/capacity impairment (34)

Medical conditions that need ax [sic; assessment]/may be causing the [sic] insomnia (38)

This theme fell into four subthemes: sleep-disordered breathing, complex health/neuromuscular conditions, other sleep disorders, and complex mental health.

#### Sleep-disordered breathing

Participant responses indicated a strong preference to exclude those with symptoms suggestive of comorbid sleep disordered breathing (SDB), particularly OSA. While this was noted as a potential inclusion criterion (see ‘Other’ relevant comorbidities above), there was a preponderance of responses recommending SDB as an exclusion criterion, suggesting high consensus between the participants on this point. This theme also included references to commonly used screening measures of sleep disordered breathing, for example, OSA50 (Chai-Coetzer et al., 2011)., and Epworth Sleepiness Scale (ESS; Johns, 1991).

History of sleep disordered breathing, untreated (29)

Insomnia with symptoms suggesting another sleep disorder e.g. OSA (50)

ESS>16 (54)^3^

#### Complex health/neuromuscular conditions

Participants recommended that other complex health and neuromuscular conditions should be considered as exclusion criteria, as should the presence of other medications (e.g. pain medications for chronic pain).

Complex medical history (31)

Respiratory conditions (56)

Neuromuscular disorders and signs of hypoventilation (44)

#### Other sleep disorders

Participants suggested that the presence of other sleep disorders including the presence of sleep medications, central disorders of hypersomnolence (e.g. narcolepsy), parasomnias, and sleep paralysis should prompt exclusion from the Direct to Psychology Pathway.

Insomnia without co-existing sleep disorder or symptoms suggestive of (18)

#### Complex mental health

The final subtheme under comorbidities for exclusion was the presence of complex mental health conditions including psychosis, neurodivergent presentations, inability to provide consent/capacity impairment, suicidality, and substance misuse.

Complex mental health history (31)

Significant risks e.g. suicidality (39)

#### ‘Other’ presenting issues/impressions

Other presenting issues identified by respondents as suitable exclusion criteria included those triaged as category 1, patients requiring fitness for work assessments, or seeing another Psychologist privately for sleep intervention.

Pt requiring fitness for work ax [sic; assessment] (i.e. Drivers licence) (47)

(a query of)seeing psychologist privately/for sleep intervention with other [sic; another] psychologist (48)

### Patient flow

Participants were asked to reflect on what they thought the right *Patient ‘Flow’* through the Direct to Psychology pathway should be, and three significant ‘Paths’ emerged within the consensus group – Direct to Psychology, Medical Review First, and Concurrent Pathway (patient is on the waitlists to see both Psychology and Sleep Physician concurrently). On reflecting on these pathway options, five predominant themes emerged in the confidential participant responses: Direct to Psychology pathway, Flexible pathways, Concurrent pathway, Medical Review First pathway and ‘Other’ Considerations.

#### Direct to psychology

Six responses were consistent with endorsing a Direct to Psychology pathway, whereby participants who met inclusion and exclusion criteria and entered the Psychology service at triage could have psychological intervention and be discharged from this service without seeing the Sleep Physician where indicated.

Pts [sic; patients] should be directly referred to the sleep psychology [sic] without the need to see the sleep specialist (58)

Start with Direct to Psychology referrals that are ‘obvious’ to test the system and build capacity/improve referrals/information (69)

#### Flexible pathways

A theme of remaining flexible with patient flow was present in the responses, such that patients could be sent down any of the three pathways and move between these flexibly as required.

Enter anytime insomnia identified – tx [sic; therapy] for SDB can occur concurrently (70)

#### Concurrent pathway

A theme emerged from some participant responses which was consistent with a concurrent pathway (patient is waiting to see psychology and medical concurrently, but with a likelihood that they will see psychology first).

Concurrent pathway is essential (65)

#### Medical consultation first pathway

Two responses were consistent with a theme that patients should see the Sleep Physician first before on-referral to Psychology (where Psychology is indicated at triage), with one response highlighting a risk of negative patient perception of being referred to Psychology before medical assessment.

Psychology stigma → patient perception as negative if referred to Direct to Psychology for ‘med’ [sic; medical]/sleep issue (64)

#### ‘Other’ considerations

Other considerations for patient flow centred around recommendations for endpoints (discharge) from the service, including completion of the intervention program, and provision of information to the referring external doctor on discharge.

Endpoint – completion of program → summary letter to referring Dr (74)

### Benefits

Participants were asked to reflect on their perception of the *Benefits* of a change to a Direct to Psychology Model, and two predominant themes in the responses emerged: *Service Efficiencies*, and *Improved Outcomes*.

#### Service efficiencies

Participants noted that improved service efficiencies were a likely outcome of a change to the service model. Within this theme, two subthemes were evident; streamlined processes and reduced physician demands.

More efficient use of resources (81)

#### Streamlined processes

Several responses clustered around the subtheme of streamlined processes, reduced bottlenecks to service delivery, and need for fewer (unnecessary) appointments.

Streamline care, reduce waiting times (82)

#### Reduced physician demands

Another clear subtheme was the benefits of a reduced burden on medical/physician clinics when appropriate referrals are triaged to the psychology pathway.

Less insomnia in medical clinics (77)

#### Improved patient outcomes

Participants also anticipated a benefit to patient outcomes, with two subthemes emerging; Increased access/timely care and tailored/patient-centred.

Reduced wait times for pts [patients] = reduced severity exacerbation (84)

#### Increased access/timely care

Within the increased assess/timely care subtheme, participants anticipated reduced wait times for medical and psychology services and increased capacity for psychology generally within the multidisciplinary model because of the service model redesign.

Improve access/build capacity (83)

See health professional faster (87)

#### Tailored/patient centred

Appropriateness of services and a patient centred approach to care decisions was also evident as patient outcomes benefit subtheme.

More tailored approach to meeting their health requirements (88)

### Risks

Finally, participants were asked to identify potential Risks of a change to a Direct to Psychology Model, and to provide suggestions for remediating these. Four predominant themes emerged: Gaps in Assessment/Treatment, Reduced Access/Efficiencies, ‘Other’ Potential Risks and No Risks.

#### Gaps in assessment/treatment

Respondents identified a risk of gaps in assessment and treatment with a Direct to Psychology redesign, with two key subthemes emerging; missed medical diagnoses, and not enough information for appropriate triage.

Pts missed/undiagnosed comorbidities would benefit less from solo psychology (require other MDT input) (96)

#### Missed medical diagnoses

The potential to miss medical diagnoses if patients were referred Direct to Psychology was the preeminent subtheme to emerge for potential risks of the service model change, particularly concerning undiagnosed sleep-disordered breathing. A recommendation to mitigate this was that patients should complete screening measures to assist with identifying potential comorbidities. We note that these suggested measures are not all currently included in a standard battery of screening tools utilised by primary care in referring into the service other than the Epworth Sleepiness Scale (Johns, 1991).

Missing co-morbid sleep disorders – OSA 50/STOPBANG/ESS for all pts (97)

Might miss other health concerns if sleep physician not seeing patient first (98)

#### Not enough information for appropriate triage

Participants also identified that referrals to the service contain varying degrees of relevant information that could inform what pathway patients may be indicated for.

Referrals don’t contain enough info for appropriate triage or wrong triage (95)

#### Reduced access/efficiencies

Whilst improved access and efficiencies were identified as likely benefits of the service model redesign, this also emerged as a theme for potential risks to consider in planning for the service change. Within this theme, two predominant subthemes were evident: slower access to medical treatment, and increased psychology demand.

Wait times – increase in psychology without additional FTE [sic; full-time employee] (especially when referrers aware of MOC and psychology services available) (93)

#### Slower access to medical treatment

A potential risk identified was that patients may not receive medical treatment as quickly if referred to psychology only.

Not treated as quickly if referred onto psychology only (92)

#### Increased psychology demand

A subtheme around the risk of increased psychology demand without an increase in resources was a potential risk to be considered and mitigated within the service model redesign.

Over supply of patients (99)

#### ‘Other’ potential risks

Participants identified other potential risks to consider with a Direct to Psychology model, referencing the potential for stigma associated with referral to psychology if a patient expected to be sent to a medical professional, and equity considerations around access to technology and patient baseline health literacy.

Don’t want referrals held up based on inequity of access to tech[nology]/health literacy (63)

#### No risks

Finally, a subtheme suggestive of no risks associated with the MOC redesign was identified and was associated with an assumption of flexibility within the system, such that patients can access the medical pathway as required.

Nil – still able to refer back to medical pathway for opinion etc (94)

### Summary of Round-Robin and clarification

The round-robin stage and subsequent clarification generated several team recommendations in response to each of the initial 3 questions asked of participants. These responses are summarised below, and the raw responses as transcribed with the team during the workshop are presented in Supplemental Item 4.0.

#### Question 1: Inclusion criteria

The round-robin responses were consistent with a consensus of inclusion criteria of a referral for insomnia or symptoms of insomnia, high insomnia severity scores, or referral to psychology generally. The team also identified questions of diagnosis of anxiety or other psychological conditions, comorbid insomnia and sleep-disordered breathing, and relapsed patients who had previously engaged with psychology as suitable for inclusion to the pathway. Re-referral if intolerant to CPAP therapy was initially identified, however on discussion, the team reached agreement this would only be suitable if sleep-disordered breathing had been already re-assessed for any changes and for CPAP titration.

#### Question 2: Exclusion criteria

The round-robin and clarification stages generated exclusion criteria for the Direct to Psychology pathway of category 1 (urgent referrals), neuromuscular disorders, a history of untreated sleep disordered breathing, high likelihood of OSA on established screening measures (high STOPBANG (Chung et al., 2008), OSA50 (Chai-Coetzer, 2011), Epworth Sleepiness Scale Johns, 1991)), comorbid respiratory and non-respiratory sleep conditions (such as restless legs syndrome, parasomnias, hypersomnia’s and sleep paralysis), fitness for work or other motor vehicle licensing concerns, the presence of multiple medications, and chronic pain/neuralgia on high pain medications. The team also identified complex medical and mental health histories where the health or mental health issues are unstable or unresolved should be exclusion criteria, as should acute suicidality or other mental health risks. Finally, the patient not consenting to referral to the Direct to Psychology pathway, and patients who are already receiving psychological treatment for sleep were also suggested as exclusion criteria for the pathway.

#### Question 3: Flow

On the question of patient flow, in the round-robin and clarification stage three main patient flow options were identified by the team. These were that on triage by the physician the patient could be filtered to any of three options – a medical pathway only (where patients could be referred to psychology later as per the current service model), a ‘concurrent’ medical and psychology path, and a ‘psychology only’ (Direct to Psychology) pathway where indicated patients could later be referred to the medical pathway. Entry to the psychology pathway options would occur if the psychologist accepted the referral, and the patient then consents to this triage decision. The MDT reiterated that early and clear communication and explanation to the patient of this service model change would allow patients the opportunity to accept or decline this treatment pathway option. In the psychology-only (Direct to Psychology) pathway, the MDT identified that assessment by the psychologist with key screening measures such as the Insomnia Severity Index (ISI; Bastien et al., 2001) and ESS would inform whether a referral to the medical team was indicated.

### Summarising and voting

On summarising the outcomes from the round-robin, the team identified three service model options for voting; ‘status quo’ referring to the medical pathway as per the existing service model, ‘Direct to Psychology’ whereby indicated patients are referred to psychology first with referral to the medical pathway to occur for indicated patients via the psychologist, and ‘Concurrent’ model whereby indicated patients would be referred to psychology at triage for assessment and intervention whilst concurrently awaiting a medical appointment. Participants voted on their preferred model/s, perception of the ease of implementing each model, and perception of the ease of measuring each model option. Participants could provide the same score for preference/ease of implementation/ease of measurement for more than one model option. The results of voting are presented in Table 2.

**Table 2.** Voting.

Model	Preference (0%)	Ease of Implementation (%)	Ease of Measurement (%)
Status Quo	0	60	37.5
Direct to Psychology	50	40	37.5
Concurrent Pathway	50	0	25

Participants indicated an equal preference for the Direct to Psychology or concurrent pathway options. No participants preferred to maintain the current service model. Participants indicated however that making no changes (status quo) would be easiest to implement, as no changes would be occurring. Participants indicated that Direct to Psychology would be the next easiest to implement and would be equally easy to measure/track outcomes as the status quo model. No participants indicated that the concurrent pathway would be the easiest to implement and only 25% of participant responses indicated that it would be the easiest model to measure/track change.

### Phase 2: Presentation of final models

The outcomes of the consensus groups were presented at a collaborative MDT in-service, with the ‘Concurrent’ model identified as presenting with the best benefit: risk ratio for the first round of implementation. Physicians would have access to the initial referral information from primary care referrers including demographic and medical history and the Epworth Sleepiness Scale, which would provide an initial indication of suitability for the concurrent psychology pathway. Given the wait times for patients suitable for this pathway, patients would see the psychologist first before seeing the sleep physician, and decision making about whether a medical appointment was still necessary after psychology assessment and/or intervention could be established with consultation with the patient, physician, and psychologist after initial psychological assessment and as needed throughout the patient’s journey.

**Figure 1.**
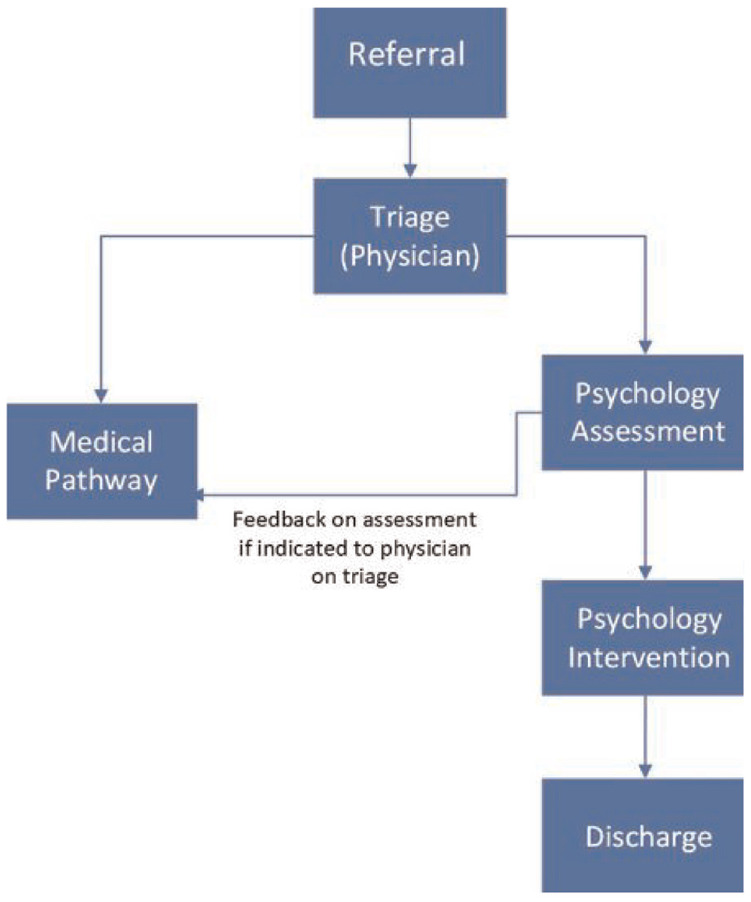
Concurrent model.

Figure 1 presents the model agreed upon by the multidisciplinary team for the psychology service including agreed inclusion and exclusion criteria. Pathways related to medical assessment and intervention, or other MDT pathways are not represented in this figure. Note that patients who were not identified at triage as suitable for concurrent referral to psychology could still be referred to the psychology service after input from the physician as per standard care. Additional information derived from Phase 2 is presented in Supplementary Item 5.0. To manage risks associated with psychology as first contact within the model, a comprehensive psychological assessment including screening tools for OSA using items from the STOP-BANG (Chung et al., 2008)and Berlin Apnoea Questionnaire (Netzer et al., 1999), hypersomnolence disorders (using items from the Cataplexy Questionnaire (Anic-Labat et al., 1999) and the presence of neuromuscular conditions, respiratory conditions and driving risk was built into the model. Patients presenting with symptoms consistent with these exclusion criteria on initial assessment with the psychologist are discussed with the triaging physician and psychological treatment is suspended if required pending physician assessment.

After the in-service, the team was satisfied with the model and inclusion and exclusion criteria and reached an agreement to commence implementation of the service model change from 1 July 2023.

**Table:** 

Inclusion Criteria (one or more): i. referred for insomnia and/or sleep psychology. ii. Suboptimal CPAP/MAS for treatment adherence intervention (if medical management optimized) Exclusion criteria (one or more): i. significant new/changed sleep-disordered breathing (or identified significant sleep- disordered breathing) ii. highly comorbid medical patients (e.g., neuromuscular conditions/weakness, respiratory conditions) iii. diagnosis of hypersomnia/question of central disorder of hypersomnolence iv. Category 1 referrals v. Driving risk

## Discussion

Sleep disorders are common and costly to society, with insomnia being the most common of these in the community with significant health, social, and economic consequences (Deloitte Access Economics, 2021; Natsky et al., 2020). Such a highly prevalent condition with significant impacts requires equitable access to evidence-based interventions. However, challenges exist in managing the serviceability of this need in the community, in particular, prolonged wait times for specialist outpatient services. Psychological services are not isolated in this challenge, and to this end other allied health disciplines have implemented innovative models of care with great success. Direct access to allied health models of care have been developed and implemented in high-demand service areas such as emergency departments (e.g. Goodman et al., 2018; Stute et al., 2018) and in post-operative orthopaedic and rehabilitation tertiary settings (e.g., Sobb et al, 2022; Morris et al., 2014).

Within sustainable healthcare, allied health-led models have demonstrated high patient, referrer, medical and allied health professional satisfaction, improved health outcomes, as well health economic cost savings (Cottrell et al., 2021; Espie et al., 2012; Mutsekwa et al., 2022). Our study sought to translate direct access models of care to a public multidisciplinary sleep service in Brisbane, Australia. The MoC designed through a consensus group with multidisciplinary team member participants indicated overall positive sentiment towards MoC redesign with a Psychology pathway.

Qualitative analysis identified several themes around potential risks of MoC change including considerations around patient safety and the management of comorbid medical conditions, as well as managing likely increased psychology demand. A subtheme suggestive of minimal risks associated with the MOC redesign was also present and was associated with an assumption of flexibility within the model. Qualitative analysis indicated that the most acceptable model was a concurrent pathway, whereby patients who meet the co-developed inclusion and exclusion criteria at triage first undertake psychology assessment and intervention before a medical appointment. The model allows for psychology-led discharge or to continue on to the medical specialist appointment as indicated. The model also allows for suspension or cessation of psychology intervention after comprehensive assessment if medical issues requiring more urgent medical review meeting exclusion criteria are identified at assessment.

Addressing participant concerns about an increase in demand for psychology was paramount to the acceptability of the proposed model. To address this, a ‘Stepped Care’ MoC which incorporates patient-led digital insomnia intervention, as well as group and trainee workforce interventions as first line, with escalation to more intensive 1:1 intervention ‘upstream’ for higher acuity presentations was also developed and implemented in the sleep service alongside the ‘Direct to Psychology’ MoC and is described in detail in an associated manuscript in preparation. We expect that on implementing these complimentary MoCs, consumer and service-level outcomes relative to the pre-implementation status quo will be substantially improved and will mitigate the risk of an increase in psychology-service demand associated with a direct access pathway.

Limitations of this study included a small participant sample size, which although not uncommon in qualitative studies, may have limited the generalisation of these findings to other hospital and health services. Due to the multidisciplinary nature of the sleep service, a traditional grounded theory approach to data collection was not possible, and as such a semi-structured approach was taken to guide participant discussion. While we believe this did not bias the data, future studies may propose a grounded theory approach to avoid any clinician-researcher apriori bias that may be present in participant-guided questions.

This implementation science study presents a methodology for achieving service reform using the resources and structures available, whilst also identifying areas for improvement. We were able to achieve consensus on a model of care redesign which included a psychology-first pathway, but future studies should innovate primary care referral practices to better screen OSA patients using validated measures such as the STOP-BANG (Chung et al., 2008), the Berlin Apnoea Questionnaire (Netzer et al., 1999), or the Sleep Apnea subscale of the Sleep Disorders Questionnaire (SDQ-SA; internal document adapted from the Berlin Apnea Questionnaire) to enable more accurate pathway decisions earlier in the process. As it stands, additional screening for comorbid conditions that would meet the pathway exclusion criteria, occur at the point of psychology first contact and assessment, which manages risk within the model but could be achieved earlier with referral process reform. Furthermore, future studies should investigate the development of an optimisation algorithm determining cut-points from valid screening measures from the point of referral, to determine triage and pathway decisions into the sleep service.

The MoC model allows for flexibility to treat psychology-indicated presentations from the outset, with the intention that once resolved, this may remove any barriers to patients engaging with medical pathway interventions, or ideally, patients are eligible for psychology-led discharge. Improved access to care and more efficient and expedited wait times were likely benefits of the service redesign and will be the focus of future publications on the implementation outcomes of this project.

## Supplemental Material

sj-docx-1-hpq-10.1177_13591053241267272 – Supplemental material for Direct to psychology for sleep disorders: Innovating models of care in the hospital and health service Supplemental material, sj-docx-1-hpq-10.1177_13591053241267272 for Direct to psychology for sleep disorders: Innovating models of care in the hospital and health service by Sara Winter, Sara Crocker, Tricia Rolls, Deanne Curtin, Jessica Haratsis and Irene Szollosi in Journal of Health Psychology

## Notes

1. AHI: Apnoea Hypopnea Index – a diagnostic tool for severity of obstructive sleep apnoea (OSA). AHI > 30 indicates severe OSA.

2. Cat 2 and 3 refers to the category assigned at triage indicating urgency of the presentation and timeframe for initial consultation. Category 3 (of 3) are triaged as ‘non-urgent’ and initial consultation should occur with 365 days of being added to the outpatient waitlist. Category 2 are ‘semi-urgent’ and should be seen within 90 days. Category 1 are ‘urgent’ and should be seen for initial consultation within 30 days.

3. Suggestive of severe excessive daytime sleepiness often associated with untreated obstructive sleep apnoea.
