# Supplementary material for: Direct to psychology for sleep disorders: Innovating models of care in the hospital and health service
Source: J Health Psychol. 2024 Aug 5;30(5):989–1003. doi: 10.1177/13591053241267272 (PMC11977826; doi:10.1177/13591053241267272)
Supplement: sj-docx-1-hpq-10.1177_13591053241267272 – Supplemental material for Direct to psychology for sleep disorders: Innovating models of care in the hospital and health service [file sj-docx-1-hpq-10.1177_13591053241267272.docx]

**Direct to Psychology for Sleep Disorders: Innovating Models of Care in the Hospital and Health Service**

**Supplementary materials**

**1.0 Questions from Phase 1 Workshop**

1. “What inclusion criteria should be included for patients to be assigned to Direct to Psychology?”

2.“What exclusion criteria should be included for patients to be assigned to Direct to Psychology?”,

3. “What do you think is the right ‘flow’ through the “Direct to Psychology Model” (in-points and end-points)”

4. “What benefits to you foresee from a change to a Direct to Psychology Model?”, and

5. “What risks to you foresee from a change to a Direct to Psychology Model and how might we mitigate these?”

**2.0 Consensus Workshop Stages**

- *Stage 1: Silent Generation:* participants individually wrote down in phases or statements their responses for up to 20 minutes.
- *Stage 2: Round Robin*: the facilitator conducted a round-robin feedback where all participants read out their responses, until no new ideas were forthcoming. All ideas are written on a virtual whiteboard for review. The first three questions in each workshop were prioritised at this step, with the two final questions on benefits and risks summarised and evaluated from the written responses (silent generation) post-workshop.
- *Stage 3: Clarification:* the facilitator encouraged group interaction including questions and comments on the listed ideas. Similar ideas, on agreement from participants, were grouped together. Example service models based on the generated ideas were developed collaboratively with the group. The facilitator hosted discussion around the generated ideas and example service models to ensure clarity and to enable group members to make an informed decision when it came to voting at Stage 4.
- *Stage 4: Summarising and Voting:* Based on results of stages 1-3, the results were summarised by the participants with guidance from the facilitator, and service model options were decided upon. Participants are asked to individually vote on their preferred models on as scale from 1 to 5 (1= least preferred model/5=preferred model). It was possible for participants to prefer more than one model equally. The preferred model/s (highest score from 1-5) for each participant was assigned a value of 1 and these votes were tallied for a total score indicating overall votes for the team’s preferred model. Where participants indicated that an equal preference for more than one model option, each option was assigned a value of 1. Participants were also asked to individually rank how achievable each example service model would be to *implement* and also to *measure* the outcomes of each service model from idea from 1 to 5 (1=extremely difficult implement to 5=easy to implement/ 1=extremely difficult to measure to 5=easy to measure). Again, the model assigned the highest value from 1-5 for each participant for each of these criteria were assigned a value of 1 each for to be tallied for the overall ‘votes’ for each model. This stage of the process was confidential.

**3.0 Coder Instructions and Coding Strategy**

Coders were instructed to work systematically through the entire data set, giving full and equal attention to each data item, and identify interesting aspects in the data items that may form the basis of repeated patterns across the data set. On identifying repeated features of the data (semantic content), coders then organised their data into meaningful groups (themes). Individual extracts of data could be coded in as many different ‘themes’ as they fit into, and any data which did not ‘fit’ into an overarching theme was grouped into ‘miscellaneous/other’. Coders then reviewed the themes they had generated to ensure that data within the themes cohered together meaningfully, whilst also ensuring that there were clear and identifiable distinctions between themes. Any revisions required to adhere to these principles of internal homogeneity and external heterogeneity were made at this stage. Once the coders were satisfied that the candidate themes were adequately refined and were a representation of the coded data, the themes were ‘named’. Coders were instructed to name the generated themes to be concise, punchy, and immediately give the reader a sense of what the theme is about. Coding was performed manually. The decision was taken not to use software to analyse the data (such as NVivo) due to the relatively small data set. Any uncertainty regarding the grouped data and generated themes were resolved by a third author (SW) resulting the final themes and subthemes derived from the dataset (See Results*)*.

**4.0 Raw verbatim data from Round Robin and Clarification:**

| **Inclusion Criteria** | **Exclusion Criteria** | **Patient Flow** |
| --- | --- | --- |
| Referral for insomnia or sx of insomnia | All cat 1 | Patient consents to Direct to Psychology |
| Referred for psychology | Neuromuscular disease | Ref 🡪sleep physician 🡪 med path; med and psychology path; psychology only path |
| Sleep physician referral with insomnia as primary referral qn | Fitness for work, licensing | Psych only path 🡪 assess 🡪med ref   - treatment |
| High isi score, sx of insomnia | Comorbid respiratory and non-respiratory sleep condition (RLS, parasomnias), hypersomnias, sleep paralysis | Access to ISI and ESS |
| ? dx of anxiety disorder, other psychology conditions | Multiple medications | Patient explained the process |
| Insomnia >14 | Respiratory conditions | Concurrent pathway (2) |
| Comorbid Insomnia/SDB | Hx sleep disordered breathing, untreated | Psychologist accepts referral |
| ~~Re-referral intolerant to CPAP??~~ | High stop bang/osa 50/ESS | Triage – letter -- pathway |
| Relapsed sleep psychology pts | Complex med hx (specific – unstable/unsorted) | Letter – Direct to Psychology identified. Fill in questionnaire, confirms pathway. |
|  | Complex Mental health hx/unstable? - acute suicidality/MH risks |  |
|  | Cat 1 |  |
|  | Already under the care of a psychologist who is seeing them about sleep |  |
|  | Pt doesn’t consent to Direct to Psychology |  |
|  | Chronic pain/neuralgia – high pain meds |  |

**5.0 Phase 2 MoC Information:**

The team decided that on psychologist review and acceptance of the referral to the concurrent pathway, and patient consent, patients would undergo a comprehensive psychological assessment according to DSM-V-tr and ICD-10 criteria and as per established professional guidelines (e.g., Ree et al., 2017). Assessment should include details of the primary sleep complaint, pre-sleep activities, nature of the sleep environment, usual sleep/wake schedule, presence of other nocturnal symptoms (e.g., respiratory, motor behaviours), nature of the patient's daytime activities and functioning. Other sleep-related symptoms, lifestyle factors and daytime consequences, detailed medical, substance, and psychiatric history (including suicide/self-harm risk assessments), and identification of current comorbid medical, substance, and/or psychiatric conditions. Patients would be screened for potential/suspected other sleep disorders, and other medical comorbidities such as congenital heart failure, atrial fibrillation, treatment refractory hypertension, type 2 diabetes, stroke, nocturnal dysrhythmias, pulmonary hypertension, and high-risk driving populations (such as commercial truck drivers).

If another sleep disorder is suspected (e.g., OSA, periodic limb movements, central disorders of hypersomnolence), or if other specialist care is required (e.g., Psychiatric evaluation) then a discussion with the Sleep Physician would occur to determine if Psychology intervention can proceed or be suspended while waiting for Physician Initial appointment. Concrete exclusion criteria at the psychological assessment point (wait for medical appointment before further psychology intervention) was determined as ESS>16 and driving risk. After the assessment process is complete, and commencement of psychological intervention is indicated, patients will undergo evidence-based psychological intervention, and on conclusion of intervention, discharged from the psychology service.

**6.0**

**Table 1.** Workshop Participant Demographics

*total number of participants who provided anonymous data

| **Demographics** | **Detail** | **N** | **Range** |
| --- | --- | --- | --- |
| Profession | Sleep Scientist  Sleep Physician  Psychologist | 6  3  3 |  |
| Years practicing | Early career <1  1-10  11-20  20+ | 1  3  4  4 | <1 - 22 |
| Sleep Disorders Centre tenure (years) | <1  1-5  6-10  11-20  20+ | 2  5  1  3  1 | 0 - 22 |
| Gender | Male  Female | 5  7 |  |
| Employment status | Part-time  Full-time | 7  5 |  |
|  | Total Participants* | 12 |  |
